# Analysis of Popular Gastroesophageal Reflux Disease Content on TikTok

**DOI:** 10.7759/cureus.62762

**Published:** 2024-06-20

**Authors:** Sheema Rehman, Wesam Almasri, Moaid Shaik, Yusra F Zakria, Neam Alazawi, Bradley J Warren

**Affiliations:** 1 Internal Medicine, Henry Ford Health System, Detroit, USA; 2 Pre-Clerkship, Oakland University William Beaumont School of Medicine, Auburn Hills, USA; 3 College of Osteopathic Medicine, Michigan State University, East Lansing, USA; 4 Gastroenterology, Ascension Providence Hospital, Southfield, USA; 5 Gastroenterology, Henry Ford Health System, Detroit, USA

**Keywords:** content creators, education, discern, tiktok, gerd

## Abstract

Researchers used the TikTok platform to investigate the quality of select TikTok educational content regarding gastroesophageal reflux disease (GERD). One hundred TikTok videos that fit the inclusion criteria were analyzed using DISCERN, a tool that evaluates the quality of consumer health information on the internet. There was no substantial difference in DISCERN scores between physicians and non-physician content creators. Nevertheless, both groups consistently scored low (<3) in areas such as providing sources of information, indicating the publication date of their sources, discussing treatment risks, and outlining potential consequences if no treatment is pursued.

## Introduction

TikTok is a social media application with over 1 billion monthly active users that has been used to create and upload videos ranging from two seconds to three minutes, covering daily vlogs, tutorials, setting trends, and educational content [1]. Due to its remarkable popularity and the opportunity to be a video-based educational tool, TikTok can be a source to help spread awareness and educate on common gastrointestinal conditions, such as gastroesophageal reflux disease (GERD) [2-4]. GERD is a common clinical pathology affecting millions of people worldwide [5-7]. Research has shown that many patients, regardless of their clinical diagnosis, do not fully understand the information the physician may provide, leaving them with many questions in order to treat or maintain their condition [8-10]. With a disease as prevalent as GERD, it is imperative to ensure that patients are receiving trustworthy education on their diagnosis [10]. This study aimed to investigate the quality of TikTok’s education content regarding GERD and identify areas to improve patient education through the popular social media platform.

## Materials and methods

We searched TikTok using the hashtag #GERD and examined the top videos recommended by the TikTok search algorithm throughout December 2023. Our analysis focused on identifying and including 100 videos that met specific criteria, excluding those not in English, unrelated to GERD, or duplicative of previously assessed content. To evaluate the selected videos, two independent reviewers employed DISCERN, a 16-item questionnaire that gauges the quality of consumer health information. Using a rating scale ranging from 1 to 5, where 1 indicates poor quality and 5 indicates excellent quality, the criteria assessed references, treatment risks, treatment benefits, and information relevance [11]. 

Furthermore, the videos were stratified based on five different categories: type of content creator (non-physician, physician with an MD or DO degree, private company, and other), gender (male, female, and other gender designation), physician specialty (gastroenterology and family medicine), and video types (treatment product advertisement, treatment-home remedy, information relating to GERD, personal anecdote, and other). After identifying and evaluating the 100 videos, Cohen’s kappa test was conducted to determine the inter-rater reliability.

## Results

We identified 116 potential videos, out of which 100 meet the inclusion criteria. These 100 videos collectively garnered 992,036 likes and 4,010 comments, with an average DISCERN score of 3.22, and demonstrated high inter-rater reliability (Cohen’s kappa > 0.8). When categorized by content creator, non-physician content creators accounted for 53 videos (53%), with a mean DISCERN score of 2.98. In comparison, physician content creators contributed 42 videos (42%), with a mean DISCERN score of 3.52 (Table 1). Both physicians and non-physicians received relatively low DISCERN scores (<3) in aspects such as providing information sources within the video, indicating the publish date of information sources, discussing treatment risks, and addressing potential consequences if no treatment was pursued. For completion, an analysis was done to compare different gender designations of the content creator, especially if it was a physician who created the content, as well as the video type.

**Table 1:** Statistics regarding TikTok videos by content creator, gender, physician, and video type GERD, gastroesophageal reflux disease

Categories		Number of videos	Mean number of likes	Mean number of comments	Mean DISCERN scores
Content creator	Non-physician	53	8733	248	2.98
	Physician	42	9450	343	3.52
	Private company	3	1092	21	3.00
	Other	2	64,496	591	3.5
Gender	Male	49	15,509	417	3.47
	Female	48	4819	173	2.98
	Other gender designation	3	264	6	3.00
Physician	Gastroenterology	31	10,283	375	3.4
	Family medicine	3	13,664	539	3.5
Video type	Treatment product advertisement	7	13,586	324	3.86
	Treatment-home remedy	18	13,306	414	3.81
	Information relating to GERD	51	11,657	298	3.48
	Personal anecdote	20	4340	222	2.30
	Other	6	459	39	1.83

## Discussion

Overall, there was no significant difference regarding DISCERN scores between physicians and non-physician content creators (p < 0.82). However, both groups score considerably low (<3) in providing sources of information, mentioning the published date to their source of information, discussing treatment risks, and what can occur if no treatment is pursued. A lack of significant difference between physician and non-physician content creators is a crucial factor to discuss, considering that physicians are at the forefront of providing education to patients on their diagnoses and treatment plans [12]. Addressing these deficits within educational videos by both physician and non-physician content creators can improve the quality of video information concerning GERD, as well as improve community education on GERD [11]. 

Deficits seen within the TikTok videos that lost ratings on DISCERN were providing information sources, indicating the publish date of information sources, discussing treatment risks, and addressing potential consequences if no treatment was pursued. These discussion points help viewers receive a better all-around picture of GERD, providing information through reliable sources as well as therapeutic options to treat the disease they are seeking information on. Providing only an overview of GERD but not providing any further details, such as pathophysiology or treatment of GERD, can leave the viewer with more questions than when they initially watched the video, missing crucial components needed to educate the viewer. Even in videos where there was treatment discussion, they were highly biased. Physicians provided patient testimonies or home remedies that were reported to have worked with their patients but did not disclose any further information on how these treatments relieved GERD symptoms. It is important to address treatments when educating the community on GERD, as well as backing the information with reliable sources to effectively address any questions the viewer may have.

Study limitations include a need for more significant representation between different physician groups to conduct statistical analyses, as there were only three videos by family medicine physicians, compared to 31 videos by gastroenterologists. Primary care has a wider reach to patients, and providing educational content on social media can improve education on topics such as GERD [13]. Additionally, DISCERN was originally validated to assess the quality of written educational material and has only recently been used to appraise social media video quality [14]. For future studies, a tool to assess social media content could provide a link between high DISCERN scores and viewership of the given content [15]. Lastly, TikTok’s main purpose is not an educational platform, and it is important to recognize its other purposes (funny skits, comedic content, dancing videos, etc.) if effort is given to improve educational video content quality on the social media platform [15].

## Conclusions

This study highlights the potential of TikTok as a platform for disseminating educational content on GERD but underscores significant deficiencies in the quality of information provided. While both physician and non-physician content creators are utilizing TikTok to share information about GERD, the overall quality, as measured by the DISCERN criteria, is suboptimal. Crucial areas needing improvement include providing sources of information, indicating the publication date of sources, discussing treatment risks, and addressing potential consequences of untreated GERD. These shortcomings limit the educational value of the content and could leave viewers with unresolved questions. Given the significant reach and influence of TikTok, addressing these gaps by enhancing the reliability and comprehensiveness of the content can substantially improve public education on GERD. Future efforts should focus on developing tailored tools for evaluating social media health information and encouraging healthcare professionals to contribute high-quality, evidence-based content. Recognizing TikTok’s dual role as an entertainment and educational platform is essential to balance engaging delivery with informative substance, ultimately improving community health literacy on GERD and potentially other medical conditions.
